# Embryonic Origin and Subclonal Evolution of Tumor-Associated Macrophages Imply Preventive Care for Cancer

**DOI:** 10.3390/cells10040903

**Published:** 2021-04-14

**Authors:** Xiao-Mei Zhang, De-Gao Chen, Shengwen Calvin Li, Bo Zhu, Zhong-Jun Li

**Affiliations:** 1 Laboratory of Radiation Biology, Laboratory Medicine Center, Department of Blood Transfusion, The Second Affiliated Hospital, Army Military Medical University, Chongqing 400037, China; mayxmzhang@tmmu.edu.cn; 2 Institute of Cancer, The Second Affiliated Hospital, Army Military Medical University, Chongqing 400037, China; degaochen@tmmu.edu.cn; 3 Neuro-Oncology and Stem Cell Research Laboratory, Center for Neuroscience Research, CHOC Children’s Research Institute, Children’s Hospital of Orange County (CHOC), 1201 West La Veta Ave., Orange, CA 92868, USA; 4 Department of Neurology, University of California-Irvine School of Medicine, 200 S Manchester Ave., Ste 206, Orange, CA 92868, USA

**Keywords:** tumor-associated macrophages (TAMs), heterogeneity, origins, subclonal evolution, metabolism, cancer target therapy, therapy-resistant

## Abstract

Macrophages are widely distributed in tissues and function in homeostasis. During cancer development, tumor-associated macrophages (TAMs) dominatingly support disease progression and resistance to therapy by promoting tumor proliferation, angiogenesis, metastasis, and immunosuppression, thereby making TAMs a target for tumor immunotherapy. Here, we started with evidence that TAMs are highly plastic and heterogeneous in phenotype and function in response to microenvironmental cues. We pointed out that efforts to tear off the heterogeneous “camouflage” in TAMs conduce to target de facto protumoral TAMs efficiently. In particular, several fate-mapping models suggest that most tissue-resident macrophages (TRMs) are generated from embryonic progenitors, and new paradigms uncover the ontogeny of TAMs. First, TAMs from embryonic modeling of TRMs and circulating monocytes have distinct transcriptional profiling and function, suggesting that the ontogeny of TAMs is responsible for the functional heterogeneity of TAMs, in addition to microenvironmental cues. Second, metabolic remodeling helps determine the mechanism of phenotypic and functional characteristics in TAMs, including metabolic bias from macrophages’ ontogeny in macrophages’ functional plasticity under physiological and pathological conditions. Both models aim at dissecting the ontogeny-related metabolic regulation in the phenotypic and functional heterogeneity in TAMs. We argue that gleaning from the single-cell transcriptomics on subclonal TAMs’ origins may help understand the classification of TAMs’ population in subclonal evolution and their distinct roles in tumor development. We envision that TAM-subclone-specific metabolic reprogramming may round-up with future cancer therapies.

## 1. Facts

1. TAMs mainly manifest protumoral activities, including tumor initiation, angiogenesis, metastasis, drug resistance, and antitumor immunosuppression.
2. TAMs predominantly derive from circulating monocytes with bone marrow hematopoietic stem cell (HSC) origin. This concept was not questioned until the embryonic origin of tissue-resident macrophages (TRMs) was discovered.
3. CCL2/C-C chemokine receptor type 2 (CCR2) signal axis is the most crucial chemokine signaling pathway on which monocyte recruitment depends.
4. The local proliferation of macrophages is supported by cytokines, such as CSF-1, IL-34, GM-CSF, and Th2 cytokines, including interleukin (IL) 4 and IL-13, and regulated by transcription factors, such as MafB, cMaf, Gata6, and retinoid X receptors (RXRs). These biomarkers help track down TAM subclonal evolution.
5. Metabolism and metabolites play crucial roles in shaping the phenotype and function of TAMs, especially under the framework of M1/M2 TAM activation. M1-TAM-like TAMs have antitumor activity, whereas M2-TAM-like phenotypes disrupt antitumor immunity and promote tumor growth. Therapeutics can be designed to balance the ratio of M1/M2 TAMs in a tumor.
6. Spatiotemporal single-cell transcriptomics and single-cell tracing technology offer the potential to target the subclonal evolution of cancer development in patients.

## 2. Open Questions

1. How to tear off the heterogeneous “camouflage” in TAMs by single-cell transcriptome to track down subclonal evolution to conduce to target de facto protumoral TAMs efficiently?
2. How to identify in vivo the molecular targets and determinants of driver mutations for each subclone of TAMs?
3. How to determine potential therapeutic manipulation related to diagnosis and prognosis of single-cell-based spatiotemporal subclonal evolution?

## 3. Background and Introduction

Macrophages are widely distributed in tissues and organs. Under normal physiological conditions, macrophages mainly play the “role of the scavenger in phagocytosis of senescent cells [1].” Meanwhile, macrophages perform diverse functions, including antigen presentation, inflammation initiation, regression, and tissue homeostasis under disease states [2,3,4]. It is well known that macrophages are an extremely heterogeneous population due to these cells’ plasticity and versatility in response to microenvironmental cues. On the one hand, macrophages’ specific function is about tissue-specific signals [5]. For example, microglia support neuronal circuit development in the central nervous system, while liver Kupffer cells (KCs) scavenge blood particles and dying red blood cells in the liver. On the other hand, macrophages undergo specific activation into functionally distinct phenotypes in response to immune stimuli. Based on T helper types 1 (Th1) and 2 (Th2) immune response, immunologists have established the concept of M1/2 macrophage to reflect inflammatory progressive and regressive states [6].

Besides the education by the microenvironment, the ontogeny of macrophages also contributes to their heterogeneity. Increasing shreds of evidence in mice have shown that macrophages do not originate solely from bone marrow hematopoietic stem cell (HSC)-derived monocytes [7]. On the contrary, most tissue-resident macrophages (TRMs) originate from the yolk sac and fetal liver and seed in tissues earlier than the first generation of HSCs [7,8,9]. These TRMs can self-maintain in situ throughout adult life and are restrictedly contributed by HSC-derived circulating monocytes, whose contribution differs according to organs and increases with age [2,3,4]. Recently, using single-cell RNA sequencing (scRNA-seq), Bian et al. identified two distinct HSC-independent waves of macrophages in human embryos that correspond to those seen in mice, supporting the multiple origins of TRMs [10]. It is worth noting that embryonic-derived macrophages have a distinctly different transcriptomic and epigenetic identity from their HSCs-derived counterparts [11]. Meanwhile, they perform different functions in situations such as tissue homeostasis, infection, and tissue injury [12,13,14]. Thus, it is integral to consider macrophages’ ontogeny when characterizing macrophages’ subsets in disease progression.

In tumors, macrophages are known as tumor-associated macrophages (TAMs). Under continuous acclimation to the tumor microenvironment (TME), TAMs mainly manifest protumoral activities, including tumor initiation, angiogenesis, metastasis, drug resistance, and antitumor immunosuppression [15]. TAMs are thought to be a predominantly M2-like phenotype disrupting antitumor immunity and promoting tumor growth, whereas M1-like TAMs have an antitumor activity that efficiently recognizes and destroys tumor cells through phagocytosis and cytotoxicity (reviewed by others [16]). The high M1/M2 ratio among TAMs is associated with a favorable prognosis [17]. Resetting TAMs toward the M1 phenotype has been considered a potential antitumor therapy [18,19]. However, recent research studies using scRNA-seq in human gliomas, colorectal cancer, breast cancer, and endometrial cancers have revealed that TAM phenotypes are much more complex and cannot be explained entirely by M1/M2 binary states [20,21,22]. Thus, we need more perspectives for TAM heterogeneity, which may help to target de facto protumoral TAMs. In recent years, with the new paradigm of macrophage ontogeny, it has been confirmed that both embryonic TRMs and adult bone marrow monocyte-derived macrophages (MDMs) constitute the TAM pool in tumor tissues, and the functional heterogeneity of TAMs caused by origins is gradually being revealed [23,24]. Dissecting the contributions of different macrophage origins to the TAM pool and the mechanism in functional characteristics differing from origins helps uncover TAM heterogeneity’s complete picture.

Metabolic adaptation is an essential part of macrophages’ survival and plasticity, instrumental to their specific function in diverse tissues and diseases [25]. TAMs develop and settle in tumor tissues, where they adapt to different metabolic environments to provide protumor activities. Metabolic intervening in TAMs offers a promising way to suppress their protumor activities [26]. Some excellent reviews have focused on the metabolism of TAMs and the metabolic crosstalk between the tumor cell and TAMs [27,28,29]. Recent evidence indicates that macrophages with different origins have metabolic defects or bias in homeostasis and infection. This realization raises exciting questions about the metabolic difference between TAMs from different origins and their influence on the functional heterogeneity of TAMs. Focusing on these questions, we will review recent discoveries on TAM origins and TAM metabolic characteristics and discuss the effects of ontogeny on TAM metabolism and advances in targeting TAMs for cancer therapy.

## 4. TAM Origins

**Figure 1:**
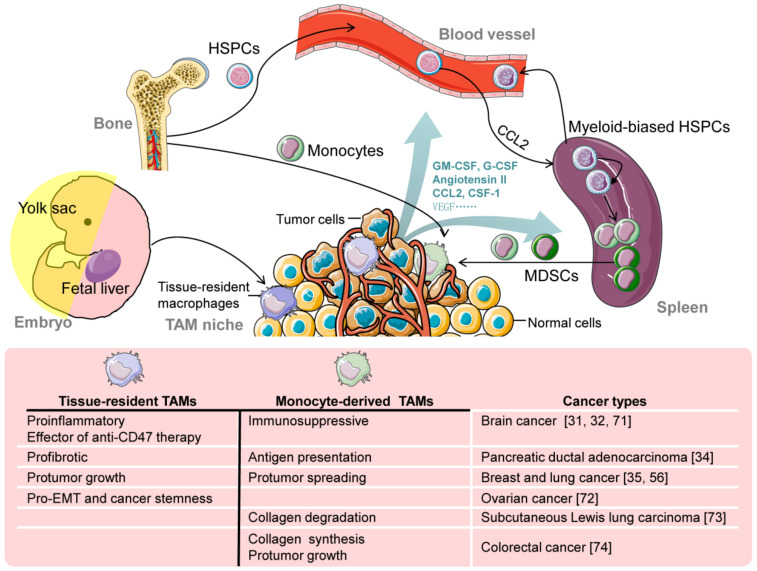
The origin and functional heterogeneity of tumor-associated macrophages (TAMs). There are two main origins of TAMs: tissue-resident macrophages (TRMs) and hematopoietic stem and progenitor cell (HSPC)-dependent monocytes. Most TRMs originate from the yolk sac and fetal liver. Under steady-state conditions, TRMs self-maintain in situ throughout adult life and are restrictedly contributed by circulating monocytes from HSPCs, depending on organs and increases in age. Monocyte-derived TAMs originate from not only bone marrow HSPCs but also splenic myeloid-biased HSPCs induced by cancer. Tumor-derived factors mediate the expansion and differentiation of myeloid-biased HSPCs and the recruitment and differentiation of monocytes and myeloid-derived suppressor cells (MDSCs). Tissue-resident TAMs and monocyte-derived TAMs (moTAMs) have different functions in tumor progression. EMT: epithelial-mesenchymal transition; HSPCs: hematopoietic stem and progenitor cells; MDSCs: myeloid-derived suppressor cells; TAMs: tumor-associated macrophages; TRMs: tissue-resident macrophages.

Tumors arise from normal cells through genetic alterations gaining unlimited growth capacity, but tumors are more than masses of cancer cells. Massive infiltration of immune cells is a feature of tumors, including macrophages and their precursors [30]. The prevailing view for a long time held that TAMs predominantly derive from circulating monocytes with bone marrow HSC origin. This concept was not questioned until the embryonic origin of TRMs was discovered. Accumulating evidence suggests that, besides circulating monocytes, embryonic TAM is a critical and non-negligible source for heterogeneous TAMs (Figure 1). The coexistence of embryonic TAMs and MDMs has been confirmed in a variety of tumors, such as brain tumor [31,32], breast tumor [33], pancreatic ductal adenocarcinoma (PDAC) [34], lung cancer [35], hepatocellular carcinoma (HCC) [36], and colon adenoma [37]. Based on the coexistence of TAMs with different origins, two new problems arise: (1) the contributions of different origins to the TAM population and (2) the distinct roles of TAMs from different origins in tumor development.

## 5. Contribution of Different Origins to TAM Population

The population of TAMs is contributed by the local proliferation of tissue-resident TAMs and the recruitment and differentiation of circulating monocytes. There is no doubt that the TAM niche regulates both proliferation and recruitment. The macrophage niche is the microenvironment around them, constructed from the surrounding cells, extracellular matrix, and cytokines [38,39,40]. The TAM niche’s composition and functional characteristics change continuously as the constantly reshaping of TME during tumor progression. Furthermore, the TAM niche’s attributes may exist among different types of tumors, particularly concerning the tissue’s intrinsic properties where the tumor is located.

On the other hand, in a specific tumor, the TAM niche’s characteristics may be different as the anatomic localization at the level of subtissue, such as the hypoxic zone and perivascular zone, tumor islands, and stromal regions [41]. The local proliferation of macrophages is supported by cytokines, such as colony-stimulating factor 1 (CSF-1), IL-34, granulocyte-macrophage colony-stimulating factor (GM-CSF), and Th2 cytokines, including interleukin (IL) 4 and IL-13 [42,43], and regulated by transcription factors, such as MafB, cMaf, Gata6, retinoid X receptors (RXRs) [44,45,46]. On the other hand, their contributions to proliferation are tissue and context-specific [47]. In a tumor, tumor-derived CSF-1 and GM-CSF’s critical role in TAM proliferation is recognized [48,49]. Whether other determinants affect TAM proliferation and whether their contributions are tumor type-specific remains to be further studied.

Circulating monocytes are known to be produced from the myeloid lineage differentiation of hematopoietic stem and progenitor cells (HSPCs) in the bone marrow and differentiate irreversibly into macrophages or dendritic cells (DCs) upon tissue entry [49]. The recruitment of circulating monocytes into the tumor is due to chemotactic signals released from tumor cells or nonmalignant stromal cells, including cytokines, chemokines, complement components, and so on [50,51,52,53]. These chemotactic signals act as more than chemoattractants; they also act as survival and differentiation signals. Besides being involved in monocyte recruitment, CSF-1 can promote local proliferation and alternative activation of TAMs [37,54,55]. Among these chemotactic signals, the CCL2/C-C chemokine receptor type 2 (CCR2) signal axis is the most crucial chemokine signaling pathway on which monocyte recruitment depends and is widely used a target in TAM research and cancer treatment [56]. Research has found that bona fide undifferentiated monocytes reside in the resting spleen and outnumber circulating monocytes [57]. Splenic reservoir monocytes can mobilize quickly into blood circulation, depending on angiotensin II in response to inflammation [57]. A tumor is associated with abnormal hematopoiesis in the bone marrow and extramedullary tissues, especially the spleen [58,59]. Myeloid-biased HSPCs accumulate in the spleen and replenish splenic monocytes and produce myeloid-derived suppressor cells (MDSCs) continuously [60]. Besides, monocytic MDSCs, one subset of MDSCs, can further differentiate into TAMs, which are another source of TAMs in the framework of MDMs [61,62]. Tumor-induced splenic accumulation of myeloid-biased HSPCs is related to the recruitment of circulating HSPCs via the CCL2/CCR2 axis and the subsequent local education by splenic stromal cells [63].

Additionally, enhanced myeloid-biased HSPCs are observed in patients’ peripheral blood with various tumors [64]. Further, tumor-induced splenic erythroid progenitor cells fail to differentiate into mature red blood cells, which may indirectly favor the myeloid-biased differentiation [58]. Tumor-derived factors, including GM-CSF, G-CSF, angiotensin II, mediate the expansion and differentiation of splenic myeloid-biased HSPCs into monocytes, MDSCs, and TAMs [64,65,66]. It is worth noting that HSPCs from the bone marrow in tumor-bearing mice are not myeloid biased [63]. It is unclear whether there are phenotypic and functional differences in TAMs derived from splenic monocytes and bone marrow monocytes. In addition to the changes in gene expression, what are the prevalent gene mutations within TAMs? Is their activity/phenotype altered predominantly due to signals from other cells? To address these questions, Wang et al. explored how APOBEC3B (A3B), a cytidine deaminase, contributes to the APOBEC mutation pattern in hepatocellular carcinoma (HCC) [67]. A3B and A3BE68Q/E255Q-expressed proteins interact with polycomb repressor complex 2 (PRC2) and regulate H3K27me3-mediated chemokine CCL2 to recruit massive TAMs and MDSCs, suggesting a deaminase-independent role of the A3B in modulating the HCC microenvironment. The prevalence rate of gene mutations at a specified point in time or over a specified period of time may be defined by ongoing developing spatiotemporal single-cell transcriptomics and single-cell tracing technology, which offer subclonal evolution of cancer development in patients.

In different studies, the relative content of MDMs and TRMs in the TAM pool has significant variability. This variability may be related to the tissues and subtissues where the tumor is located. As shown in Figure 1, the origin and functional heterogeneity of TAMs might provide a glimpse into novel therapeutic directions. Tumor-associated macrophages (TAMs) play pivotal roles in tumor progression and metastasis, but the contribution and regulation of different macrophage populations remain unclear. In the mouse model of orthotopically inoculated hepatic Hepa1-6 tumors, which were derived from myeloid-specific NOTCH blockade by the conditional disruption of the recombination signal binding protein Jκ (RBPj cKO), Ye et al. found that one population, namely, embryonic-derived Kupffer cell (KC)-like TAMs (i.e., tentatively named KC-like TAMs (kclTAMs)), dominated over another population, namely, monocyte-derived TAMs (moTAMs). The kclTAMs came with more than 90% [36], while CCR2-independent TRMs accounted for less than 20% in the early stage of colon adenoma [37]. More interestingly, Ccr2 −/− mouse RBPj cKO kclTAMs increase the proliferation via enhanced β-catenin-dependent WNT signaling, while moTAMs are genetically blocked, suggesting liver specificity for RBPj cKO kclTAMs. This difference may be due to the abundance of embryonic-derived KCs in the liver tissue, while TRMs in the colon tissue are regularly replaced by MDMs after birth [7].

In the lung, the relative proportion of resident interstitial macrophage (IM)-derived TAMs and MDMs is associated with tumor development’s anatomical site because resident IMs are abundant in the pleura airways and at the periphery of large blood vessels [35]. The composition of TAM pools also varies with the volume and progression stage of the tumor. This variation is tissue-specific. The increasing abundance of CCR2-independent TRMs was observed in conjunction with colon adenoma progression due to CSF-1 depending on self-renewal [37]. However, there was no difference in the relative contents of MDMs and TRMs between small and large tumors in glioblastoma (GBM) [32]. Irradiation could significantly increase the abundance of MDMs in GBM due to inflammation and the blood–brain barrier disruption. Recently, single-cell mapping of the tumor microenvironment of patients with primary brain tumors or brain metastases revealed that the immune response to cancer in the brain is shaped by cancer type, with metastases favoring MDM invasion gliomas characterized by activated microglia [68].

Given that there is a lack of specific and canonical molecular markers or lineage tracking systems for TAM subtype identification, the relative contents of MDMs and TRMs in the TAM pool may be associated with the method of subtype identification. For example, using Flt3–Cre lineage tracing, microglia-derived TAMs composed about 65% of the bulk TAM population in glioma, while using CX3CR1GFP/WT; CCR2RFP/WT knock-in mice, over 85% of the TAMs within the glioma are thought to be derived from inflammatory monocytes [31,32]. Collectively, these studies suggest that the composition of TAMs from different origins depends on tumor type, subtissular location, progression stage, and stimuli-like irradiation and inflammation.

## 6. Functional Heterogeneity of TAMs from Different Origins

In GBM, the multiple genetic lineage tracing models were used to track the ontogeny of TAMs and demonstrated that resident microglia and circulating monocytes jointly contribute to TAMs in murine [31]. Gene expression profiles of TAMs, regardless of their origins, were distinct from those of normal microglia or blood monocytes. This feature is consistent with the notion that macrophages undergo in situ reprogramming in TME [69]. Moreover, resident microglia-derived TAMs and MDMs possess distinct gene expression patterns [31]. For example, MDMs express higher levels of gene transcripts involved in immunosuppressive functions, such as IL-10, CCL17, and CCL22.

In contrast, microglia-derived TAMs are enriched for the expression of CCL4 and tumor necrosis factor-α (TNF-α), chemokines associated with a proinflammatory response. The differential gene expression patterns suggest unique functions for each TAM subset in tumor progression. Later, another study confirmed the coexistence and differential gene expression patterns of TRMs and MDMs in GBM: genes associated with proinflammatory cytokines and metabolism were enriched in microglia-derived TAMs [32]. Besides, the authors found that MDMs predominate within the GBM parenchyma, preferentially locating to perivascular regions, suggesting the extravasation of monocytes through blood vessels within the tumor. In contrast, microglia-derived TAMs primarily reside at the tumor periphery, corresponding to the fact that resident microglia do not migrate easily through the brain tissue [70]. The upregulation of the antiphagocytic (i.e., “do not eat me”) surface protein CD47 is a well-characterized immune evasion mechanism by tumor cells. Microglia-derived TAMs are effector cells of tumor cell phagocytosis in response to anti-CD47 blockade [71]. In PDAC, significant portions of TAMs are found to originate in embryonic development and expand via in situ proliferation during tumor progression [34]. These embryonic TRMs promote PDAC progression. Although circulating monocytes are also crucial for sustaining the TAM population, they are dispensable for PDAC progression, inconsistent with studies in breast cancer and GBM [31,33]. Embryonic TRMs in PDAC have a distinct profibrotic phenotype, while MDMs have a higher expression of molecules involved in antigen presentation [34]. In the omentum, embryonic CD163+ Tim4+ TRMs express a unique transcriptional profile and have a causative role in the metastatic spread of ovarian cancer by promoting epithelial-mesenchymal transition (EMT) and a cancer stem cell-like phenotype of tumor cells [72]. In the lung, accumulation of embryonic resident interstitial macrophages (IMs), not alveolar macrophages (AMs), is mainly correlated with tumor growth in vivo, while MDMs are associated with tumor spreading [35]. In breast cancer, CCR2+ MDMs also facilitate tumor metastasis [56]. Extracellular matrix degradation and remodeling are essential for tumor growth and metastasis. Simultaneously, CCR2^+^ MDMs are involved in synthesizing the collagenous matrix in orthotopic colorectal cancer and the degradation of the collagenous matrix through mannose receptor-dependent cellular uptake in subcutaneous Lewis lung carcinoma [73,74]. Together, these studies indicate that TRMs have distinct functional phenotypes and anatomical locations for MDMs, and the contributions of TAM subtypes to tumor progression maybe vary with tumor types. Further studies are needed to determine whether embryonic TRMs accumulate in more other types of tumors, their accurate molecular markers, roles in tumor progression, and relationship with prognosis.

## 7. TAM Metabolic Reprogramming

In TME, the proliferation and apoptosis of tumor cells arise simultaneously, and the apoptotic cell clearance mainly depends on the process of engulfment of dying cells, namely, efferocytosis [75] of TAMs, which means that TAMs are constantly taking up endogenous and exogenous materials, including cells and molecules. It is clear that these processes closely affect the metabolism of TAMs. Furthermore, metabolism and metabolites play crucial roles in shaping macrophages’ phenotype and function, especially under M1/M2 activation [76]. Metabolic reprogramming is needed for TAMs in response to TME cues and adapts to the nutritional needs of tumor cells and supports the survival of TAMs themselves. Given the prevailing view about the M2-like phenotype of TAMs, we next discuss the recent findings in TAM metabolism based on the metabolic remodeling in M1/M2 activation.

## 8. Arginine Metabolism

The original definition of mammalian M1 and M2 macrophages is derived from observations that promote Th1 and Th2 inflammatory responses, respectively, with differences in their arginine metabolism [77]. M1 macrophages metabolize L-arginine to L-citrulline and nitric oxide (NO) via inducible nitric oxide synthase (iNOS), whereas M2 macrophages upregulate arginase 1 (ARG1) to metabolize L-arginine to L-ornithine, which is a precursor for polyamines and proline components of collagen, an essential element in tissue repair [78]. ARG1, as the maker of M2 macrophages, is highly expressed in TAMs within hypoxic regions, far from the vasculature, derived by the synergistic role of hypoxia and lactate on the activation of MAPK/ERK signaling in mouse models of pancreatic neuroendocrine and breast tumors [79]. These TAMs within hypoxia regions express higher vascular endothelial growth factor A (VEGF-A) levels than TAMs located in other tumor regions, contributing to angiogenesis and faster-growing tumors. Redirecting TAMs’ arginine metabolism from polyamine synthesizing to nitric oxide synthesizing by supplementing sepiapterin could inhibit immunosuppression and growth of mammary tumors [26], suggesting a new approach to cancer management.

## 9. Glucose Metabolism

As the first substrate of glycolysis, glucose is transported into TAMs for ATP generation. Glucose consumption varies with different environmental stimuli. Classically activated M1 macrophages undergo a metabolic rewiring, including enhanced anaerobic glycolysis, blocking of the tricarboxylic acid (TCA) cycle, and the inhibition of mitochondrial respiration. Alternatively, activated M2 macrophages are characterized by enhanced fatty acid oxidation (FAO, also known as β-oxidation) and oxidative phosphorylation (OXPHOS), recently reviewed in detail by Ryan [80]. Research has shown that enhanced glucose utilization is also a metabolic characteristic of M2 macrophages induced by IL-4, even though the glycolysis is less than that in M1 macrophages induced by interferon-γ (IFN-γ) plus lipopolysaccharide (LPS) [81]. In M1 macrophages, glucose-derived pyruvate is converted mainly to lactate due to the inhibitions of pyruvate dehydrogenase (PDH), aconitase 2, and electron transport chain by NO [82].

On the other hand, M2 macrophages exhibit mitochondrial pyruvate import to fuel the TCA cycle and support FAO and OXPHOS [81]. Increased glucose metabolism is associated with mTOR complex 2 (mTORC2)-mediated upregulation of interferon regulatory factor 4 (IRF4) and activation of the AKT–mTORC1 pathway in M2 macrophages [81,83]. The inhibition of glycolysis by 2-deoxyglucose (2-DG), a competitive inhibitor of hexokinase II (HK2), is reported to impair M2 activation [81,83]. Based on the research results using 2-DG, glucose metabolism is proposed to be critical for M2 macrophage differentiation. However, the off-target effect of 2-DG was subsequently found to question this conclusion. 2-DG impairs OXPHOS, in addition to inhibiting glycolysis, resulting in the suppression of ATP production and failure of JAK–STAT6 activation and M2 polarization [84]. JAK–STAT6 activation is ATP dependent and plays a crucial role in M2 polarization triggered by IL-4.

Meanwhile, neither glucose depletion nor glycolysis interference with galactose inhibits M2 macrophage differentiation as long as OXPHOS is intact [84]. These findings indicate that glycolysis is not a requirement for M2 macrophage differentiation. Nevertheless, there are two significant functions of glycolysis in M2 macrophage activation. First, glycolysis-produced ATP can activate the JAK–STAT6 pathway induced by IL-4 during OXPHOS inhibition. Second, pyruvate produced from glycolysis can fuel acetyl-coenzyme A (CoA), which enters the TCA cycle to support OXPHOS and acetylate histones to regulate some M2 gene expression [83,85]. The conversion of pyruvate to acetyl-CoA needs the PDH complex, which is negatively regulated by pyruvate dehydrogenase kinase 1 (PDK1). The knockdown of PDK1 enhances pyruvate flux to acetyl-CoA and promotes M2 macrophage activation [86].

**Figure 2:**
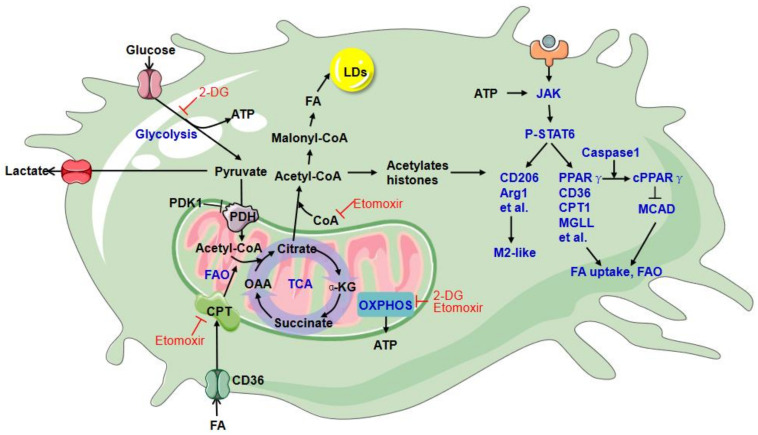
Glucose and lipid metabolism in TAMs. TAMs exhibit a pronounced glycolytic signature enhanced lipid uptake and accumulation. Glucose-derived pyruvate is partly converted to acetyl-CoA by the PDH complex to fuel the TCA cycle, supporting OXPHOS. ATP, from both glycolysis and OXPHOS, can be used to activate the JAK–STAT6 pathway in M2-like activation. 2-DG inhibits TAM phenotype and function due to impaired OXPHOS in addition to glycolysis. Activation of the JAK–STAT6 pathway induces gene expression related to FAO and FA uptake. The caspase1-mediated cleavage of PPARγ attenuates MCAD activity, and FAO promotes LD formation. The elevated level of the scavenger receptor CD36 plays a crucial role in lipid uptake and accumulation in TAMs. The LD formation is essential for TAMs as a stable source of fatty acids for fatty acid oxidation (FAO). Acetyl-CoA converted from citrate feeds FA synthesis and acetylates histones to regulate TAM gene expression. Etomoxir, an inhibitor of CPT1, inhibits FAO and inhibits complex I of the electron transport chain and depletes CoA, eventually impairing M2 activation and the protumor growth activity of TAMs. 2-DG: 2-deoxyglucose; αKG: α-ketoglutarate; CoA: coenzyme A; CPT: carnitine palmitoyltransferase; FA: fatty acid; LD: lipid droplet; MCAD: medium-chain acyl-CoA dehydrogenase; MGLL: monoacylglycerol lipase; OAA: oxaloacetate; OXPHOS: oxidative phosphorylation; PDH: pyruvate dehydrogenase; PDK1: pyruvate dehydrogenase kinase 1; TAMs: tumor-associated macrophages; TCA: tricarboxylic acid.

A recent report showed that human melanoma cell-educated TAMs are highly glycolytic, in turn, to maintain their M2-like polarization characterized by increased expression of the markers CD206, CD301, and CD163 [87]. Human PDAC cell-educated TAMs also exhibit a pronounced glycolytic signature, and 2-DG can disrupt the pro-metastatic phenotype of TAMs by reversing TAM-supported angiogenesis, extravasation, and epithelial-to-mesenchymal transition of tumor cells [88]. In vivo, increased glycolysis is observed in monocytes purified from tumor tissues compared with monocytes isolated from the paired blood or nontumor tissues of patients with HCC [89]. Glycolysis activation in peritumoral monocytes induces immune privilege via the PFKFB3/PD-L1 axis and is responsible for tumor-induced upregulation of TNF-α, IL-10, and IL-1β in monocytes [89]. These results indicate that glucose metabolism is enhanced in TAMs and plays a critical role in TAMs’ phenotype and function (Figure 2). However, all these research studies applied 2-DG to disrupt glycolysis. As discussed above, in M2 macrophage activation, 2-DG impairs OXPHOS, inhibiting glycolysis, and glycolysis inhibition alone by glucose depletion does not inhibit M2 macrophage differentiation. Further enhanced glycolysis in TAMs by REDD1 (regulated in development and DNA damage response 1) deficiency-mediated mTORC1 activation causes tumor vessel normalization by competing for glucose with endothelial cells, preventing vessel leakiness, hypoxia, and metastases [90]. Thus, enhanced glycolysis’s contribution to the phenotype and function of TAMs needs further study in vitro and in vivo. In summary, reported M2 markers include CD115, CD206, PPARG, ARG1, CD163, CD301, Dectin-1, PDL2, and Fizz1, while genetic markers associated with M1 polarization include IL1a, IL1b, IL6, NOS2, TLR2, TLR4, CD80, and CD86.

## 10. Lipid Metabolism

Besides glycolysis activation, enhanced fatty acid uptake and FAO are other salient features of M2 macrophages. Enhanced FAO is dependent on IL-4-induced STAT6 activation and then upregulates genes related to fatty acid uptake and oxidation, such as peroxisome proliferator-activated receptor-γ (PPARγ), PPARγ coactivator-1b (PGC-1b), CD36, carnitine palmitoyltransferase I (CPT1), medium-chain acyl-CoA dehydrogenase (MCAD), and so on [91]. The CPT system is critical in β-oxidation of long-chain fatty acids and transports long-chain acyl-CoA into mitochondria, consisting of one transporter and two mitochondrial membrane enzymes: CPT1 and CPT2 [92]. Etomoxir is a widely used specific inhibitor of CPT1 to explore the biological roles of FAO. Research has shown that etomoxir could inhibit IL-4-induced M2 macrophage activation and also inhibit the M2-like phenotype of TAMs and their protumor activity [91,93], suggesting the critical role of FAO for M2 activation and TAM function. However, etomoxir’s multiple off-target effects are gradually being confirmed, including inhibition of complex I of the electron transport chain [94] and induction of oxidative stress [95]. Using genetic models to knock out CPT1 or CPT2 in macrophages both result in disability of performing FAO but do not affect IL-4-induced M2 activation [96,97]. Additionally, the off-target effect to deplete CoA by excess etomoxir is demonstrated to be responsible for blocking IL-4-induced M2 activation, which is independent of CPT1/2 expression and FAO [97]. These results call for genetic models and optimal etomoxir concentration to inhibit FAO with a minimal off-target effect.

Furthermore, inhibitions of the complexes of the electron transport system and ATP synthase by inhibitors do not block IL-4-stimulated M2 activation [97], suggesting that the role of OXPHOS in M2 activation is not necessary. This finding is consistent with the idea that cytoplasmic ATP produced by glycolysis is enough to support IL-4-induced activation of JAK–STAT6 signaling and M2 phenotype when OXPHOS is inhibited [84]. In contrast, OXPHOS is necessary for the M2 phenotype during glycolysis inhibition [84]. Based on the current studies’ results, enhanced glycolysis and OXPHOS are redundant in IL-4-induced M2 polarization.

As known, M2 activation contains distinct subtypes with different functions due to stimulation cues: M2a (induced by exposure to IL-4 and IL-13) and M2b (activated by immune complexes, toll-like receptors (TLRs), and apoptotic cells) drive Th2 responses. At the same time, M2c (deactivated by glucocorticoids, transforming growth factor-β (TGF-β), or IL-10) participates in immune suppression and tissue remodeling [6]. In contrast to IL-4-stimulated M2 macrophages, anti-inflammatory macrophages triggered by apoptotic cells’ engulfment are reported to require FAO and OXPHOS, which is verified by siRNA targeting to CPT1/2 in addition to etomoxir [98]. The engulfment of apoptotic cells induces the reprogramming of M2-like TAMs to some extent [99,100]. From this, we reasonably speculate that FAO and OXPHOS may be indispensable in these apoptotic-cell-mediated TAMs but dispensable in IL-4-dependent TAMs. IL-4 might be derived from intratumoral CD4^+^ T lymphocytes [101]. Given the complexity of TME, the roles of FAO and OXPHOS in TAM reprogramming may vary with diversified stimulating factors.

Interestingly, fatty acid uptake and subsequent lipolysis seem to be essential for M2 macrophage activation and TAM generation (Figure 2). Both CD36 and lysosomal acid lipase (LAL) are upregulated and critical for full M2 activation of macrophages in response to IL-4 [102]. Increased lipid accumulation has been found in TAMs from human tumor tissue, including breast tumor, prostate tumor, colon cancer, and melanoma, compared with macrophages in normal tissues [103]. The elevated level of the scavenger receptor CD36 plays a crucial role in lipid uptake and accumulation in TAMs and is associated with FAO and OXPHOS to generate more energy for TAMs. Impairing lipid uptake by CD36 deficiency can block the generation of TAMs by suppressing the activation of JAK–STAT6 signaling and abolish TAMs’ protumor growth activity [103]. The caspase1-mediated cleavage of PPARγ translocates to mitochondria and attenuates MCAD activity and FAO, thereby leading to the accumulation of lipid droplets (LD) in TAMs [104]. Besides, monoacylglycerol lipase deficiency (MGLL) contributes to lipid overload in TAMs [105]. LD is the storage form of excess fatty acids and a stable source of fatty acids for FAO. Disrupting the formation of LD could attenuate fatty acid uptake-induced OXPHOS and ATP production, as well as the expression of CD206 and immunosuppressive capacity in TAMs; increase cytotoxic CD8^+^ T cell tumor infiltration, and inhibit tumor growth [106]. In summary, lipid metabolism is critical in the phenotype and function shaping of TAMs.

## 11. Metabolic Regulation of TAM Ontogeny

We discussed that TAMs from different origins, or ontogeny, have other tumor development functions in the previous parts. In contrast, the phenotype and function of TAMs are intrinsically linked to metabolism. So far, most studies have focused on the collective metabolism of TAMs and M2-like metabolic reprogramming. The heterogeneity of TAMs, which cannot be explained entirely by M1/M2 binary states, calls for more perspectives on metabolic studies of TAM subtypes. Next, we reviewed the macrophages’ metabolic and functional differences from different origins and discussed TAM ontogeny’s metabolic regulation.

**Figure 3:**
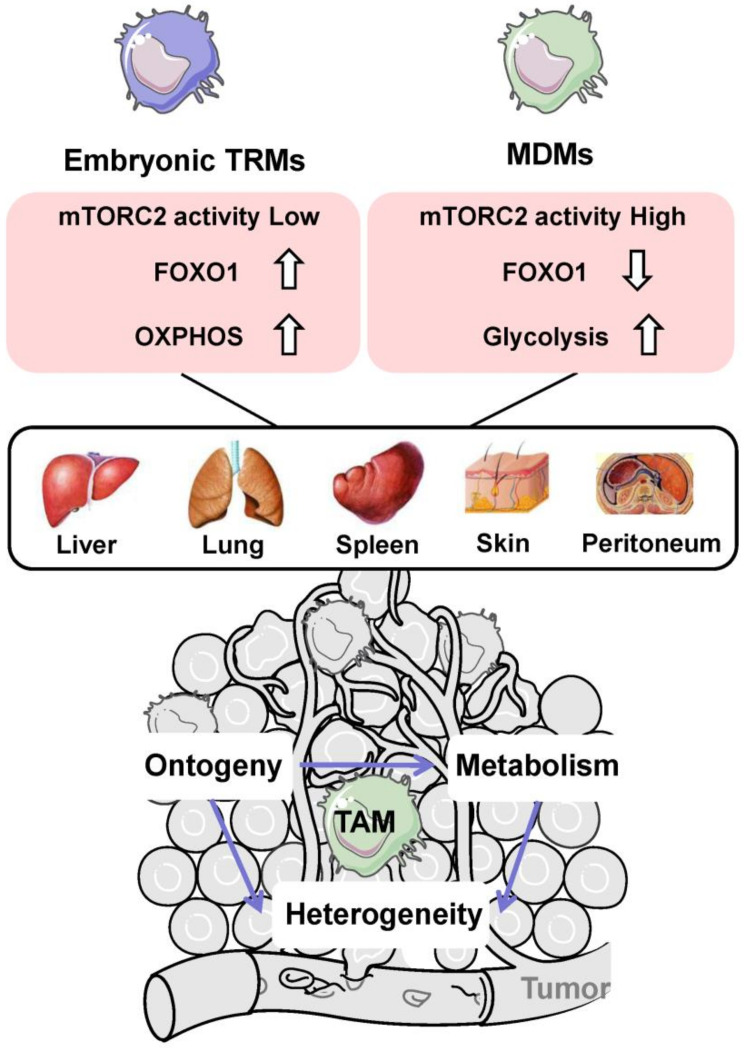
The metabolic regulation of TAMs’ ontogeny. Embryonic TRMs in the peritoneum display decreased glycolysis and increased OXPHOS compared with monocyte-derived peritoneal macrophages due to mTORC2/FOXO1 axis. Higher FOXO1 expression is also observed in other TRMs (in the lung, spleen, skin, and liver) compared with MDMs, indicating this metabolic bias’s tissue universality. Embryonic TRMs and bone marrow MDMs constitute TAM pools in various tumor types. The metabolic imprints in TAMs from different origins may play an essential role in TAM heterogeneity. TRMs: tissue-resident macrophages; MDMs: monocyte-derived macrophages. TAMs: tumor-associated macrophages.

Accumulating evidence suggests that macrophage ontogeny has metabolic differences to determine their function (Figure 3). It is reported that fetal monocytes possess increased mitochondrial bioenergetics and glycolytic capacity to promote their survival and expansion, which allow them to outcompete primitive yolk sac-derived macrophages in the development of lung AMs [107]. Cord blood-derived macrophages show incomplete activation in responding to IFN-γ and IL-10 due to defective glycolysis, compared with adult peripheral blood-derived macrophages [108]. Given that cord blood contains rich primitive HSCs, the main precursors of cord blood-derived macrophages. At the same time, monocytes are macrophage precursors in adult blood, and this metabolic difference may be imprinted in macrophage ontogeny. Tissue-resident peritoneal macrophages display increased OXPHOS and decreased glycolysis compared with monocyte-derived peritoneal macrophages, and these metabolic features are, in part, mediated by the mTORC2/FOXO1 axis [109]. Notably, higher FOXO1 expression is also observed in other TRMs (in the lung, spleen, skin, and liver) compared with MDMs within the same organ, suggesting that metabolic differences between TRMs and MDMs may be tissue universal [109]. Consistent with this, resident AMs in the lung do not exhibit classical glycolytic reprogramming in response to LPS, and glycolytic inhibition does not affect their ability to produce inflammatory cytokines, in contrast to infiltrating MDMs [110]. Recently, tissue-resident AMs have also been found to be much less responsive to IL-4 and helminth infection than IMs due to impaired glycolysis [111].

Similarly, in *Mycobacterium tuberculosis* infection, AMs are upregulated for fatty acid uptake and β-oxidation and increase the lung’s bacterial burden. In contrast, IMs are highly glycolytically active and restrict bacterial growth and survival [112]. The distinct metabolic state of AMs is thought to be conferred by the lung environment, one of the considerations being the low glucose level in the alveoli [113]. Considering that AMs derive from fetal liver monocytes, while IMs originate from yolk sac precursors and are replenished partly by MDMs in adults [114], the origin or ontogeny provides another account for the diverse adaptive immunometabolism of lung macrophage subtypes. Such findings suggest that metabolism is significant concerning macrophage ontogeny, and macrophages from different origins have distinct metabolic characteristics, especially embryonic TRMs and bone marrow MDMs.

Embryonic TRMs and bone marrow MDMs have been found in various tumor types. Although less attention has been paid to the TAM subtypes’ metabolic features, there is evidence to suggest that the metabolic difference from TAMs’ ontogeny is a vital part of TAMs’ metabolism. Performing scRNA-seq on TAMs of human gliomas, results have shown that MDMs significantly elevate levels of genes that are rate-limiting for citrate and succinate processing compared with microglia-derived TAMs, suggesting an activation of the TCA cycle in MDMs [22]. Meanwhile, these MDMs express significantly higher levels of genes typically associated with an immunosuppressive M2-like phenotype, such as *IL10* and *TGFB2*, associating with poor patient survival [22]. This is consistent with the research that limiting CCL2-derived monocyte infiltration can prolong GBM-bearing mice’s survival [32]. More lines of evidence are needed to determine whether different origins of TAMs have unique metabolic imprints and whether these various metabolic imprints are essential in developing and treating tumors.

## 12. Targeting Subclonal TAMs for Cancer Therapy

Considering the vitally essential roles of TAMs in suppressing antitumor immunity and promoting tumor progression, concerted efforts have been made to target TAMs for tumor immunotherapy. TAM-associated biomarkers include CCR2, CSF1R, MARCO, PDL2, CD40, CCL2, CSF1, CD16, and PDGF beta. Currently, TAM-targeting antitumor therapy’s principal strategies mainly include suppressing survival, proliferation, and recruitment or promoting phagocytosis or repolarization of TAMs. Numerous related therapeutic agents are under clinical trial [115,116]. Based on data from a pivotal phase 3 trial (NCT02371369) [117], in August 2019, the United States Food and Drug Administration (FDA) approved pexidartinib (PLX3397) for the treatment of tenosynovial giant cell tumors (TGCTs) [118]. Pexidartinib is a small-molecule antagonist of the CSF1 receptor and induces significant TAM depletion to delay tumor growth [119]. In a mouse study of hepatocellular carcinoma, tumor-cell-derived GM-CSF could protect TAMs from being depleted by pexidartinib [49]. In this study, pexidartinib inhibits tumor growth by repolarizing TAMs toward an M1-like phenotype [49]. In other studies, depletion of TAMs by pexidartinib alone had minor or even no effect on tumor growth and tumor-bearing mice’s survival [120,121]. However, its combination with anti-PD-1 therapy or DC immunotherapy improved curative effects by enhancing CD8 T cell migration and immune activation [120,121].

CCL2/CCR2 axis is another well-studied mechanism for TAM recruitment. Moreover, recent research has revealed that CCR2 expression on tumor cells orchestrates suppression of the immune response through impeding the infiltration and activation of cytotoxic T lymphocytes and CD103^+^ cross-presenting DCs [122]. Multiple inhibitors or antibodies against CCR2 are being tested clinically as tumor treatment [115,116]. Besides directly targeting CCR2, its intracellular chemokine signal regulator FROUNT is an alternative target to block CCR2 and CCR5 [123], another primary chemokine receptor involved in TAM recruitment [124]. Further, disulfiram (DSF), a well-known anti-alcoholism drug approved by the FDA, acts as a potent inhibitor of FROUNT to reduce TAM accumulation and tumor progression in mice, which can synergistically inhibit tumor growth with the immune-checkpoint PD-1 antibody [123]. The results indicate that DSF is a drug promising for macrophage-targeting tumor therapy.

In addition to reducing TAM accumulation, promoting the antitumor activity of TAMs is another crucial strategy for TAM-targeting antitumor therapy. CD47 molecules expressed on tumor cells interact with signal-regulatory protein alpha (SIRPα) on TAMs to prevent phagocytosis, enabling the escape of innate immune surveillance. Antibodies for CD47 (Hu5F9-G4) and SIRP1α (CC-95251 and 1H9), as well as SIRP1αFc fusion protein (TTI-621), promote phagocytosis, reducing tumor burden in numerous animal models, and are being tested in a clinical trial [125,126,127]. AO-176, the next-generation anti-CD47 antibody, is developed and well-tolerated with no adverse effects due to negligible red blood cell binding [128]. AO-176 is currently under multicenter phase 1/2 clinical investigations in advanced malignant solid tumors (NCT03834948) combined with bortezomib/dexamethasone in relapsed/refractory multiple myeloma (NCT04445701). RRX-001 is a nontoxic pleiotropic anticancer small molecule in phase 3 clinical trials (NCT03699956), including downregulating CD47 on tumor cells SIRP1α on TAMs [129]. Cyclophosphamide is a broad-spectrum antitumor drug approved by the FDA in 1959, and its mechanism in the treatment of tumors is generally attributed to its alkylation, which leads to DNA fragmentation and inhibition of DNA synthesis [130]. A recent study defined a novel mechanism of high-dose cyclophosphamide promoting macrophage phagocytosis-dependent lymphoma clearance by shifting the balance of pro- and antiphagocytic factors, which includes downregulation of CD47 on tumor cells [131].

In addition to promoting the phagocytosis of live tumor cells by blocking CD47/SIRPα interaction, inhibiting the phagocytosis of apoptotic tumor cells by TAMs is another promising tumor immunotherapy strategy. TAMs-mediated engulfment of apoptotic tumor cells not only prevents inappropriate inflammatory responses by avoiding the release of cellular contents but also induces the immunosuppressive reprogramming of TAMs [132,133]. Antibody blockade of the phagocytic receptor MER proto-oncogene tyrosine kinase (MerTK) prevents apoptotic tumor cell clearance by TAMs and mobilizes antitumor immunity via the transport of tumor-derived cyclic GMP-AMP into TAMs and the subsequent activation of STING-dependent type I IFN response [134]. MerTK blockade enhances anti-PD-1 therapy and has implications for cancer immunotherapy [134]. A MerTK-selective inhibitor, MRX-2843, is under phase 1 clinical trials in adults with advanced and/or metastatic solid tumors (NCT03510104).

Metabolic programming is closely tied to the phenotypes and functions of TAMs; thus, metabolic reprogramming by targeting the immunometabolism of TAMs is a promising strategy in controlling cancer. In fact, some approaches to target the function of TAMs promote extensive metabolic rewiring. Anti-PD-1/PD-L1 therapy is a very well-performing immunotherapy for tumors. Triggering PD-1 signaling by PD-L1 on tumor cells impedes a metabolic switch toward aerobic glycolysis, which is needed by T effector cell activation and monocyte/macrophage phagocytosis, while blocking PD-1/PD-L1 reverses these immune metabolic dysfunctions together with antitumor activity [135,136]. Recent research suggests that myeloid cell-specific PD-1 ablation more effectively decreases tumor growth than T cell-specific PD-1 ablation [137]. PD-1 deficiency in myeloid progenitors induces immunometabolic changes, increasing metabolic intermediates of glycolysis, pentose phosphate pathway (PPP), and TCA cycle, significantly elevated cholesterol, which is essential for differentiation of inflammatory TAMs and DC and promotes antigen-presenting function [137]. The transcription factor c-Maf is highly expressed in TAMs, and reduction of c-Maf inhibits TAMs’ immunosuppressive function and tumor-promoting activity, accompanied by the promotion of glycolysis and partial blocking of the TCA cycle [138]. From this line, glycolysis is essential for the antitumor activity of TAMs in vivo. Although 2-DG, which is capable of targeting glycolysis in tumor cells, inhibits the M2-like phenotype of TAMs in vitro, it may aggravate the immunosuppressive function of TAMs and hinder the activation of T effector cells in vivo, both of which might be responsible for the limited clinical effect of 2-DG [139].

To the extent that cell metabolism is essential to every cell, its inhibition may cause indiscriminate killing, not only of disease-causing cells but also of healthy cells and disease-suppressing cells. This adverse effect is one of the significant challenges in metabolic research. TAM is a double-edged sword in tumor progression and treatment; thus, precisely targeting TAMs’ metabolism to improve their antitumor activity and inhibit their protumor activity is an essential issue for targeting TAM metabolism. A recent study suggests that inhibiting the environment-context selective metabolic redundancy can inhibit pathogenic cells and simultaneously reduce toxic and side effects on other cells [140]. Besides, tumor cells and nontumor cells, including TAMs, within the tumor microenvironment show more significant metabolic heterogeneity at the single-cell level than at the bulk level [141], suggesting that the map of the subclonal evolution landscape may define specific TAMs. These studies may provide new insights into targeted TAMs’ metabolism and clinical transformation. Furthermore, the inhibitions of the complexes of the mitochondrial electron transport system using the glycolytic inhibitor 2-DG (1 mM) [86] and the ATP synthase inhibitor oligomycin [142] do not block BMDM-(IL-4)-stimulated M2 activation (96), suggesting that the role of mitochondrial oxidative phosphorylation (OXPHOS) in M2 activation as anti-inflammatory macrophages (M2) is more dependent on mitochondrial OXPHOS. Thus, metabolism is a promising target for cancer therapy, and more efforts must be made in this area. More incisive methods, such as single-cell RNA sequencing [143], comparative genomic hybridization, and whole-genome sequencing, are commonly used in clinical practice; the ontogeny and metabolism of subclonal tumor-associated macrophages will lead to manifestation for precision medicine in novel therapies against dominating tumor subclones [144]. An in vivo real-time imaging [145] tracking of TAMs will allow subclonal-based treatments to maximize efficacies and minimize adverse effects [146].

## 13. Spatiotemporal Single-Cell Transcriptomics and Single-Cell Tracking Technology Offer Subclonal Evolution of Cancer Development in Patients

In-the-field scientists have reached the consensus that the concept of embryonic origin and subclonal evolution of TAMs sounds original and appealing. Still, such an idea got lost in the published literature review, with limited technology dedicated to this concept until recently. Specifically, as we have published [143] and single-cell technology has been illustrated for subclonal tracing and characterization of COVID-19 models in vivo, we wanted to model TAMs with a similar strategy. 

First, single-cell technology has recently been applied to determine antibody responses and proliferative SARS-CoV-2-specific T cells’ capabilities in convalescent COVID-19 (Sekine et al. identified SARS-CoV-2-specific CD4+ and CD8+ T cells in convalescent individuals, exposed family members, and healthy blood donors). The single-cell technology can help to map functional and phenotypic SARS-CoV-2-specific T cell immunity across the full spectrum of exposure, infection, and disease by the first measurement of T cell perturbations in COVID-19 (e.g., the percentage of neutrophils, CD4+, and CD8+ T cells in COVID-19 cases involving children [147].

Second, single-cell technology allows single cells’ isolation with phenotypic characteristics of SARS-CoV-2-specific T cells in acute and convalescent COVID-19 [148].

Third, single-cell transcriptome analyses can gravitate toward genes of the multisystem inflammatory syndrome in children (MIS-C)—Multisystem Inflammatory Syndrome in Children and COVID-19 are distinct presentations of SARS-CoV-2) [149]. The above-derived patterning can be used to prepare for single-cell transcriptomic analyses as we previously described [143,150], also in combination with a free algorithm tool [151]; thus, we can infer genetic information from the transcriptome data to discover the subclones present and understand their genetic differences specific for SARS-CoV-2 trafficking single cells and related immune responses involved in T cells and B cells.

We hypothesize that the detailed phylogenies derived from the molecular profiling of immunity against SARS-CoV-2 can recount critical events’ history and chronology during immunity progression. Determination of the single-cell lineage tracer allows for studying the immunity model’s rates, routes, and immunity drivers. A similar study using deeply resolved phylogenies for tens of thousands of cancer cells can be traced over months of growth and dissemination [152], with the power of tracing cancer progression at a subclonal resolution and vast scale, which can be combined with TAM tracking and characterization.

## 14. Conclusions

To remove the obstacles and pitfalls in tumor immunotherapy, researchers must understand TME fully. As the dominant innate immune cell population in TME, the characteristics of TAMs need to be revisited. Through the innovation of technology in cellular developmental biology, the ontogeny of TAMs has been gradually revealed. Embryonic progenitor-derived TRMs are distinct from MDMs in the genetic and metabolic field, which provide a new perspective for us to understand the phenotypic and functional heterogeneity of TAMs. The current understanding of TAM metabolism is mainly based on the TAM-mediated crosstalk with TME and M1/M2 activation framework. We ignore the metabolic differences in ontogeny and their effects on the metabolic reprogramming of TAMs. Efforts to clarify TAM subtypes’ metabolic characteristics based on ontogeny are conducive to further research and therapeutic targeting. However, reports on TAM ontogeny’s metabolic regulation have been very scarce, that almost no drugs have been designed for this. It is worth noting that the tumor context needs to be considered since TRMs from diverse organs originate from the different embryonic days.

Additionally, the metabolic patterns of macrophages are relatively convenient to detect in vitro. By contrast, it is hard to analyze the real-time metabolic pathway and interaction of TAMs in TME in vivo. This little technological advance is stunting the progress of in situ metabolism research. Thus, paying more attention to figuring out the metabolism regulation of subclonal TAMs in ontogeny and the in-situ metabolism reprogramming of TAMs in vivo may open a new avenue for drug development with metabolic targets in TAM-related tumor immunotherapy. How to determine the potential therapeutic manipulation related to diagnosis and prognosis of single-cell-based spatiotemporal subclonal evolution remains elucidated.

## Data Availability

The materials supporting the conclusion of this review have been included in the article.

## Abbreviation

2-DG: 2-deoxyglucose; AMs: alveolar macrophages; ARG1: arginase 1; CCR2: C-C chemokine receptor type 2; CoA: coenzyme A; CPT1: carnitine palmitoyltransferase I; CSF1: colony-stimulating factor 1; DCs: dendritic cells; DSF: disulfiram; EMT: epithelial-mesenchymal transition; FAO: fatty acid oxidation; FDA: Food and Drug Administration; GBM: glioblastoma; GM-CSF: granulocyte macrophage colony-stimulating factor; HK2: hexokinase II; HSCs: hematopoietic stem cells; HSPCs: hematopoietic stem and progenitor cells; IL: interleukin; IMs: interstitial macrophages; iNOS: inducible nitric oxide synthase; IRF4: interferon regulator 4; KCs: Kupffer cells; LAL: lysosomal acid lipase; LD: lipid droplet; LPS: lipopolysaccharide; MDMs: monocyte-derived macrophages; MDSCs: myeloid-derived suppressor cells; MCAD: medium-chain acyl-CoA dehydrogenase; mTORC1: mammalian target of rapamycin 1; MerTK: MER proto-oncogene tyrosine kinase; moTAMs: monocyte-derived TAMs; mTORC2: mTOR complex 2; NO: nitric oxide; OXPHOS: mitochondrial oxidative phosphorylation; PDAC: pancreatic ductal adenocarcinoma; PDH: pyruvate dehydrogenase; PDK1: pyruvate dehydrogenase kinase 1; PGC-1b: PPARγ coactivator-1b; PPARγ: peroxisome proliferator-activated receptor-γ; PRC2: polycomb repressor complex 2; REDD1: regulated in development and DNA damage response 1; RXRs: retinoid X receptors; scRNA-seq: single-cell RNA sequencing; SIRPα: signal-regulatory protein alpha; TAMs: tumor-associated macrophages; TCA: tricarboxylic acid; TGCTs: tenosynovial giant cell tumors; TGF-β: transforming growth factor; Th1: T helper type 1; TLRs: toll-like receptors; TME: tumor microenvironment; TNF-α: tumor necrosis factor-α; TRMs: tissue-resident macrophages; VEGF-A: vascular endothelial growth factor A.
